# Material Characterization of a Magnetorheological Fluid Subjected to Long-Term Operation in Damper

**DOI:** 10.3390/ma11112195

**Published:** 2018-11-06

**Authors:** Dewi Utami, Ubaidillah, Saiful A. Mazlan, Fitrian Imaduddin, Nur A. Nordin, Irfan Bahiuddin, Siti Aishah Abdul Aziz, Norzilawati Mohamad, Seung-Bok Choi

**Affiliations:** 1 Malaysia-Japan International Institute of Technology, Universiti Teknologi Malaysia, Jalan Sultan Yahya Petra, 54100 Kuala Lumpur, Malaysia; udewi2@live.utm.my (D.U.); amri.kl@utm.my (S.A.M.); nurazmah.nordin@utm.my (N.A.N.); irfan.bahiuddin@ugm.ac.id (I.B.); aishah118@gmail.com (S.A.A.A.); mnorzilawati@gmail.com (N.M.); 2 Mechanical Engineering Department, Faculty of Engineering, Universitas Sebelas Maret (UNS), Jl. Ir. Sutami 36A, Kentingan, Surakarta, 57126 Central Java, Indonesia; fitrian@ft.uns.ac.id; 3 National Center for Sustainable Transportation Technology (NCSTT), 40132 Bandung, Indonesia; 4 Department of Mechanical Engineering, Vocational College, Universitas Gadjah Mada (UGM), Jl. Yacaranda Sekip Unit IV, 55281 Yogyakarta, Indonesia; 5 Department of Mechanical Engineering, Inha University, 253, Yonghyun-dong, Namgu, 22212 Incheon, Korea

**Keywords:** MR fluid, long-term operation, double-ended damper, magnetic field, rheological properties, morphology observation

## Abstract

This paper investigates the field-dependent rheological properties of magnetorheological (MR) fluid used to fill in MR dampers after long-term cyclic operation. For testing purposes, a meandering MR valve was customized to create a double-ended MR damper in which MR fluid flowed inside the valve due to the magnetic flux density. The test was conducted for 170,000 cycles using a fatigue dynamic testing machine which has 20 mm of stroke length and 0.4 Hz of frequency. Firstly, the damping force was investigated as the number of operating cycles increased. Secondly, the change in viscosity of the MR fluid was identified as in-use thickening (IUT). Finally, the morphological observation of MR particles was undertaken before and after the long-term operation. From these tests, it was demonstrated that the damping force increased as the number of operating cycles increases, both when the damper is turn on (on-state) and off (off-state). It is also observed that the particle size and shape changed due to the long operation, showing irregular particles.

## 1. Introduction

It is known that magnetorheological (MR) fluid belongs to one of the smart materials featuring the magnetic field-dependent rheological properties and, hence, control performance of various application systems in which MR fluid is used as carrier fluid medium can be achieved by simply controlling the magnitude of the field intensity [1,2,3,4]. More specifically, MR fluid behaves like a Newtonian fluid (fluid-like phase) in the absence of the magnetic field, but its behavior rapidly changes to the non-Newtonian fluid (solid-like phase) by applying the magnetic field [5,6,7,8,9]. This special characteristic of MR fluid can provide a quiet, simple, and rapid-response interface between electronic controls and mechanical systems [10]. So far, numerous application devices and systems, such as automotive damper, have been investigated, and some of the systems are used in the practical environment. Some of the specific applications include truck seat damper [11], a prosthetic knee joint [12], and fan clutches [13]. Although these devices or systems have shown great performances, some problems were not evident in the initial phase, and need to be addressed at a later time. One of the major problems is the phenomenon called in-use-thickening (IUT), where the fluid is permanently damaged after long-term operation [11]. The IUT phenomenon frequently happens when the MR fluid is subjected to high shear rate and high shear stress over time. The original low viscosity MR fluid will thicken and change into an unmanageable paste-like material. Therefore, under these circumstances, MR fluid is no longer suitable for controlling the performances of the applied devices and systems [6,11,14,15].

The early IUT problem was observed in the truck seat damper, type Motion Master™ RD-1005 made by Lord Corporation, Cary, NC, USA [11]. The damper was continuously operated under 1200 N force with a frequency of 1 Hz, 25.4 mm stroke length, and 1 A of applied current to generate the magnetic field, for 600,000 cycles. The damper’s operation was periodically varied between on-state and off-state conditions, by applying a certain current during the observation. The result revealed that the initial force (off-state) increased more than 100% after the operation: from 200 to 500 N. The initial force increase caused by the viscosity increment of MR fluid is not acceptable for the performance of the damper, since it reduces rider’s comfort. For the case of the prosthetic knee joint MR device, it is generally known that the proper performance should last for 3,000,000 cycles [12]. However, the IUT effect has made MR fluid unusable once the device reaches 1,000,000 cycles. The prosthetic knee joint is one of the applications of MR fluid operated under direct shear mode [15]. This work identified that the knee joint stiffness increased with increasing of operation time and number of cycles. Such problems occurred as the off-state torque force of the knee joint increased in long-term operation until, eventually, the knee joint needed to be replaced due to a decrease in effectiveness. The degradation of the knee joint effectiveness is likely related to the degradation of the MR fluid caused by the IUT phenomenon. Other studies also reported similar IUT problems with most MR devices and systems after long-term operation applications [16,17,18]. Of interest is a study conducted on long-term operation of an MR fan clutch, which operated for 540 h to observe the change in MR fluid characteristics [13]. The study focused on analysis of the iron particles within the MR fluid. Three samples were observed during the durability test: one before the test, one after 108 h, and the last one after 540 h. The result showed that the torque of the fan decreased by 15% for both 3000 and 5000 rpm at the end of 540 h. Through the observation, it was identified that the magnetic particles (irons) in the MR fluid were oxidized over the time. Further observations indicated that the surface of the magnetic particles showed porous layers and, appeared rough as the result of the oxidation process. Thus, oxidation of the magnetic particles can be considered another issue that needs to be resolved in MR device application and systems subjected to long-term operation.

The above studies showed the significance of material characteristics of MR fluid when it is utilized for a long time as the main function for controlling force or torque in diverse applications. In other words, the durability or reliability of MR fluid itself plays a critical role in the lifetime operation of MR applications. For example, an MR device could not be used when the problems of IUT and particles oxidation of MR fluid occurred, even though the devices might still in good condition. More importantly, the performances of MR devices are critically affected by MR fluid condition. As commercialization of MR applications expands, the endurance of MR products becomes a more important issue to be resolved. However, research on material characterization of MR fluid used in long-term operation is considerably rare. Consequently, the main technical contribution of this work is to experimentally investigate material properties of MR fluid used as the main carrier fluid to generate field-dependent force or torque for long-term operation. In order to accomplish this goal, MR damper operated as a flow mode is adopted and operated for a long time. After completing the operation, the MR fluid is collected, and its field-dependent viscosity tested. In addition, the particle shapes are observed via scanning electron microscope (SEM) and the surface roughness of the MR damper’s spacer bar is investigated to determine the wear effect of the iron particles. It is remarked, here, that the material characterization of MR fluid, which is operated for a long time in a flow mode MR damper, has not been reported, so far, to the best of the authors’ knowledge.

## 2. Experimental Setup

### 2.1. MR Fluid

The MR fluid used in this study is a commercial one, model MRC-C1L, supplied by CK Material Laboratory (http://www.ckmaterialslab.com), Seoul, Korea, for general applications such as dampers, brakes and shock absorbers. The fluid contains magnetic particles with the weight ratio of approximately 78%, and the principal properties of MRC-C1L are listed in Table 1.

### 2.2. MR Damper and Testing Machine

**Figure 1:**
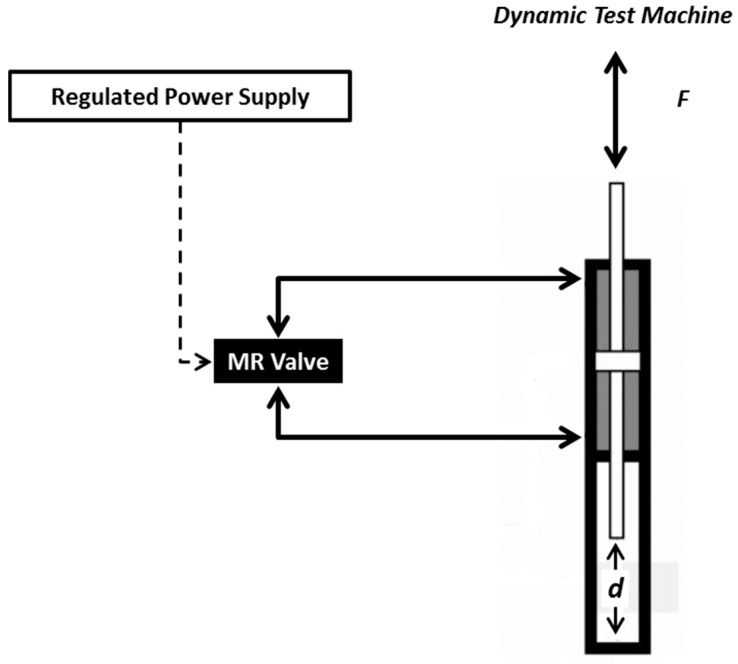
Schematic configuration of MR valve testing.

**Figure 2:**
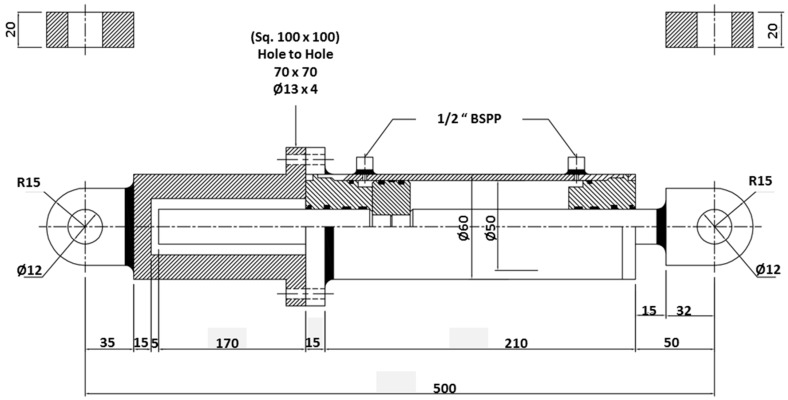
Sketch of MR damper configuration.

**Figure 3:**
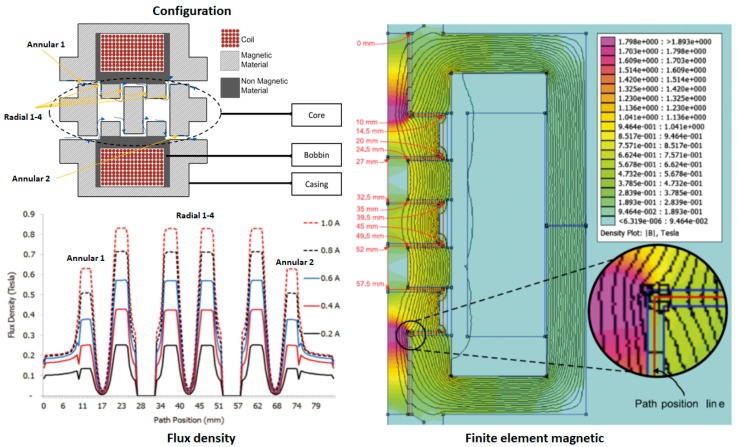
Schematic diagram for the meandering MR valve of the damper.

An experimental setup configuration for the MR valve measurement is established, as shown in Figure 1. The details of MR damper are shown in Figure 2. It was customized to accommodate the long-term operation experiment with detailed geometrical dimensions as follows: bore size = 50 mm, rod size = 30 mm, stroke length = 60 mm, and cylinder length = 500 mm. The rod and bore differ greatly in size, to emphasize volume of flowing MR fluid inside the MR damper for each stroke motion. The meandering MR valve structure of the damper is divided into three main components: casing, valve, and core. The casing, made from magnetic material, acting not only as an outer shell of the whole structure of the valve but, also, as the connector of the valve to other devices through its embedded fittings. The other functions of the casing are to guide the magnetic flux from the coil in order to minimize the loss of flux to the air, which reduces efficiency. The coil was wound around the coil bobbin, made from non-magnetic material, to generate the magnetic field in the MR valve structure. The core was used as a platform to provide an annular and radial flow channel for the path of MR fluid flow. The detailed configuration of the MR valve, including finite element magnetic simulation using FEMM, as well as magnetic flux distribution in all effective areas in the MR valve, is shown in Figure 3. The MR valve consists of 5 effective areas, namely, annular 1, radial 1 to 4, and annular 2. It can be seen that the amount of magnetic flux density between annular and radial areas are different at the same applied current. Therefore, it cannot be mentioned, definitively, the conversion of a certain current flow to the value of magnetic fields. Therefore, the magnetic flux density during an on-state experiment, Table 2 provides the amount of magnetic flux density in each region, at 1 and 2 A. This configuration allows the provision of high magnetic field strength at the fluid gaps through a combination of casing and core embedded with the bobbin, as fully described in [19].

### 2.3. Sample Characterization

The field-dependent rheological properties, such as viscosity of MR fluid (MRC-C1L), are investigated before filling the MR damper. The rheological properties of MR fluid are investigated via a rotational shear test of the parallel-plate rheometer (Anton Paar, Physica, MCR 302, Graz, Austria), at room temperature. The viscosity and shear rate of MR fluid is tested at on-state and off-state condition, using a 20 mm diameter plate (PP20/MRD/TI). The on-state condition for the viscosity and shear rate are conducted under the following conditions of the magnetic field: range from 1 A and 2 A, and sweep shear rate from 0.01 to 1000 s^−1^. From the experiment, the magnetic field of 1 A is approximately 0.19 tesla, and 2 A approximately 0.40 tesla. In addition, the magnetic particles are extracted from the MR fluid using a solvent, acetone, to characterize the morphological properties before the operation. The morphological characteristics related to size and shape of the magnetic particles in the MR fluid were observed using low vacuum scanning electron microscopy (LV-SEM), model T300 LV from Jeol, Japan, with a magnification of 3000× and 5000× at the accelerating voltage of 5 kV. Meanwhile, the corresponding composition of magnetic particles was analyzed using the energy dispersive X-ray spectroscopy (EDS) equipped in the LV-SEM. The same tests as the above mentioned were undertaken by collecting MR fluid from the damper after the long-term operation of 170,000 cycles.

### 2.4. Experimental Procedures

**Figure 4:**
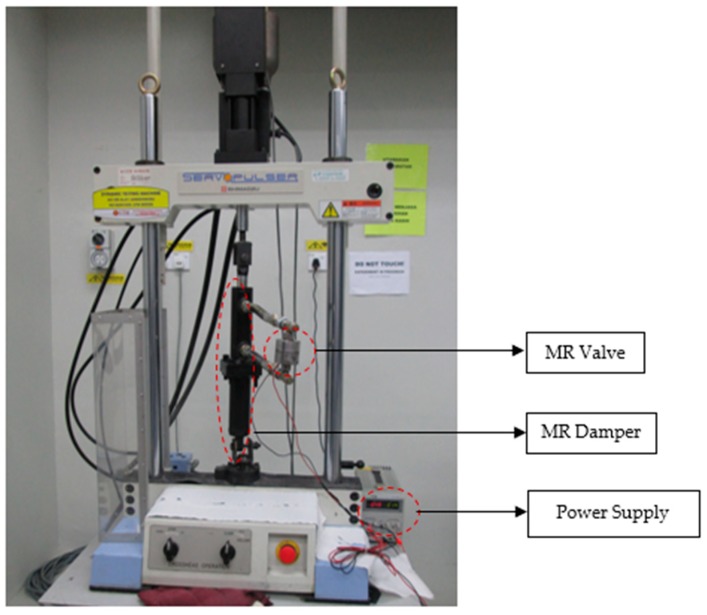
Experimental setup using fatigue dynamic test machine, including MR valve and MR damper that would be operating for 170,000 cycles.

The experimental works are conducted using the fatigue dynamic test machine, made by Shimadzu Company (Japan). The machine shown in Figure 4 is capable of generating compressed and extended forces of MR damper. MR damper is filled with MR fluid, where the damper is actuated by the dynamic test machine and the changed behavior of the fluid can be analyzed through the measured strokes of the damper. The experiment is started on the off-state condition by setting the stroke length at 20 mm with a frequency of 0.4 Hz. Then, for the on-state condition, the magnetic field of 0.5 A is continuously applied for the long operation of 170,000 cycles. The results in terms of damping force versus number of cycles are plotted to identify the change in the damping force generated from the yield stress of the MR fluid. Eventually, the applied current is cut off after the last cycle. After that, the experiment is conducted under off-state condition with a similar setup: the stroke length at 20 mm with a frequency of 0.4 Hz. The off-state damping forces before and after the long-term operation are then compared.

## 3. Results and Discussion

### 3.1. Long-Term Exposure

**Figure 5:**
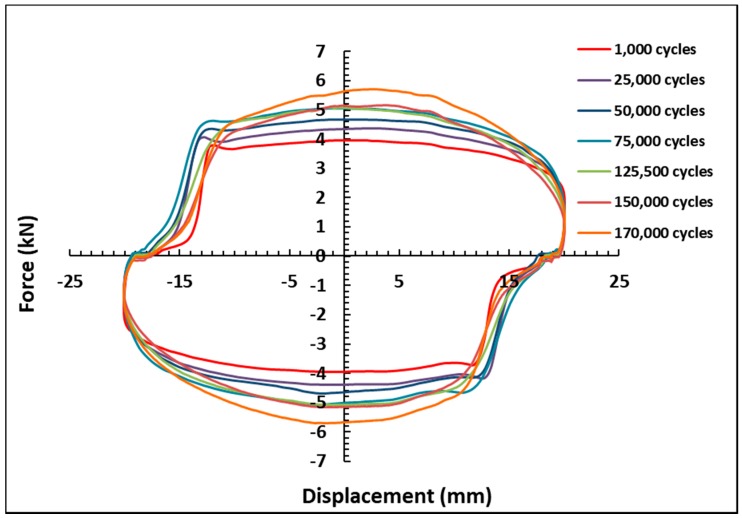
Force-displacement results at the on-state condition.

**Figure 6:**
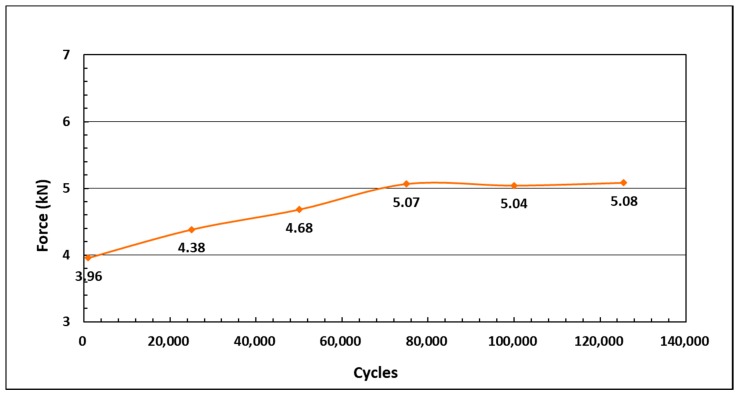
The increment of damping force with respect to the number of cycles.

**Figure 7:**
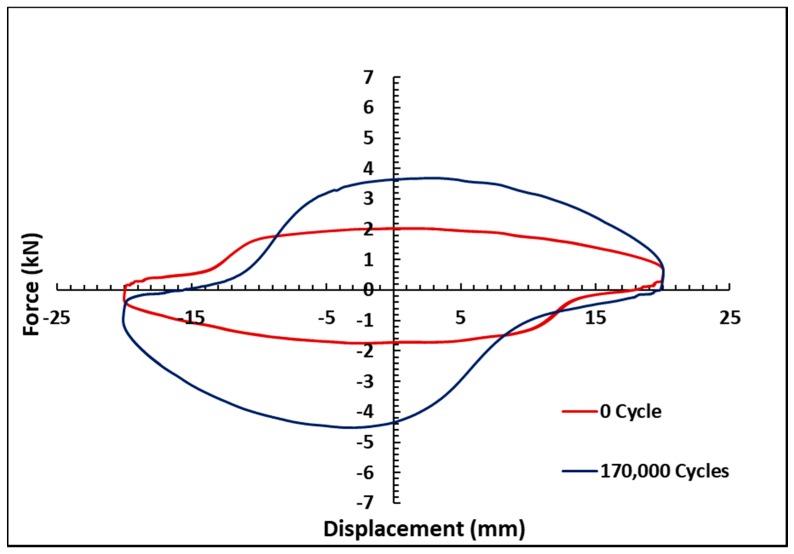
The force-displacement result in the off-state condition.

Figure 5 shows the force versus displacement graph for the on-state condition of 0.5 A as a function of the operating cycle under the fixed oscillation frequency of 0.4 Hz, and the excitation displacement amplitude of 20 mm. The influence of the number of cycles on the MR damper damping force is clearly observed from the results. In particular, the maximum damping forces are identified by 3.96, 4.38, 4.68, 5.07, 5.04, 5.08, 5.15, 5.08, 5.16, and 5.70 kN for 1000; 25,000; 50,000; 75,000; 100,000; 125,500; 150,000; and 170,000 cycles, respectively. It shows that the lowest peak force appears with the least number of cycles, while the highest force value is associated with the largest number of cycles. In general, the maximum damping forces proportionally increased with the increasing operating cycles. The phenomenon can be further observed in Figure 6. As shown in the figure, the maximum values of the damping force increase with the increasing number of cycles. The peak damping force increment, from the beginning of the operation to 170,000 cycles, is approximately 1.74 kN, or 44%, with respect to the initial peak force. This result indicates that the utilized MR fluid is affected by the long-term operation of the MR damper. In fact, from the beginning of the operation cycles to 170,000 cycles, the force increases, although, after 100,000 cycles, the force slightly decreases. Meanwhile, from 75,000 cycles to 150,000, there is no considerable difference in the force increment. The result also shows that, after 8000 cycles, the damping forces remain almost unchanged, which has revealed the damping forces’ saturation points. This phenomenon occurs when the MR fluid is continuously activated by the magnetic field for a long duration. Even though the experiment has already finished, it was assumed that the MR fluid still under the influence of magnetic field. This may be caused by the increased viscosity of the MR fluid. Figure 7 shows the force-displacement results for the off-state condition, before and after the long-term operation of the MR damper. The result indicates that the peak force increases after the long-term operation, particularly after 170,000 cycles. The initial force of MR damper is approximately 2 kN, and increases to 3.8 kN after the long operation. This increment of the initial force phenomenon indicates that the properties of the MR fluid are affected by the continuous load in a long-term operation, even for the off-state condition. This might be due to the increased viscosity of the MR fluid after the long operation. 

### 3.2. In Use Thickening (IUT)

**Figure 8:**
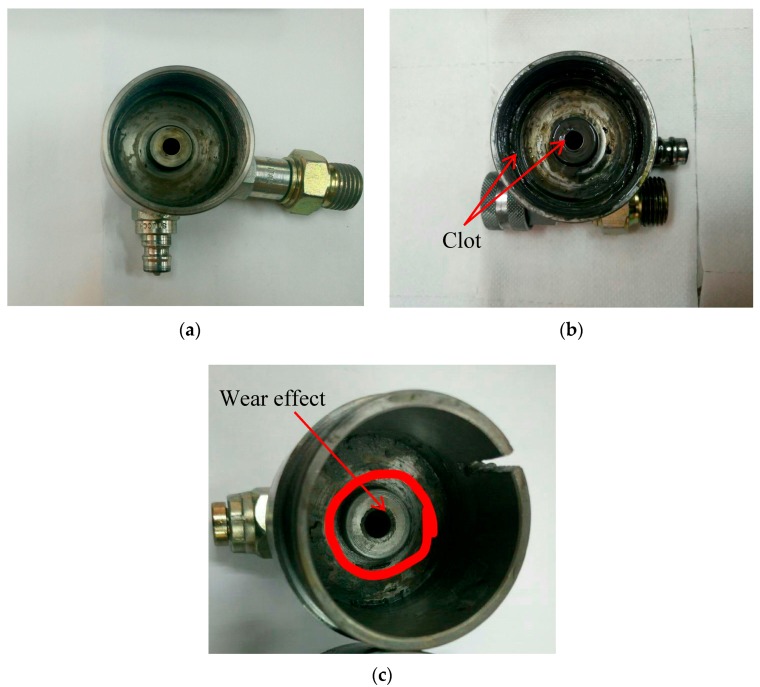
The female casing of the MR valve (**a**) before the operation, (**b**) after the long-term operation, and (**c**) the wear inside the tube.

Theoretically, as explained by Carlson [11], the viscosity of MR fluid increases during the long-term use of application that caused the IUT phenomenon. The study revealed that, with increasing usage period, the off-state viscosity also increased, resulting in an increase in the force and/or the torque of the MR application devices and systems. In this work, the same phenomenon of increasing peak MR damper force is observed in the experimental results, as shown in Figure 5 and Figure 7. In another possible scenario, although MR fluid is free to flow during the operation, some of the magnetic particles might be trapped and deposited at the MR valve. This phenomenon is also referred to as IUT. The trapped magnetic particles may cause a clot at the MR valve, interrupting the flow of MR fluid and resulting in a higher force during the longer operation process. The physical observation of the MR valve female casing is shown in Figure 8. The figure exhibits the condition of the MR valve female casing before the operation, while Figure 8b shows the same casing after 170,000 cycles and the deposited magnetic particles surrounding the valve, marked with a red arrow. Figure 8c shows the casing with the wear effect inside the tube after the long-term operation. After cleaning the casing, the wear effect can still be observed at the channel surface, along with the remaining clot that still surrounds the valve. This clot affects the MR fluid flow inside the valve by narrowing the flow channel; this is particularly evident at the tube. Therefore, the restriction of MR fluid flow causes the damping force to increase, as discussed in Section 3.1. Besides, the clot becomes thicker over time because more particles will be dumped on the channel.

The steady state model of the meandering MR valve type can be utilized to analyze the relationship between the deposited magnetic particles and the rising peak of the force. The MR valve model can be expressed by [20]

(1) $$∆P=∆P_{viscous}\left(Q\right)+∆P_{yield}\left(B\right),\;$$

where ∆P, $∆P_{viscous}\left(Q\right)$, and $∆P_{yield}\left(B\right)$ are the total pressure drop, the viscous pressure drop as a function of flow rate (Q), and the yield pressure drop as a function of magnetic field (B), respectively. Meanwhile, in general, $∆P_{viscous}$ and $∆P_{yield}\left(B\right)$ can be expressed as follows.

(2) $$∆P_{viscous}=\frac{1}{d^{3}}K_{1}Q\;$$

(3) $$∆P_{yield}=\frac{1}{d}K_{2}\tau \left(B\right)\;$$

where d is the channel gap within the MR valve, $K_{1}$ and $K_{2}$ are constants for pressure drop viscous and yield within the MR valve, respectively, and τ(B) is the yield stress as a function of the magnetic field B. While magnetic particles are deposited in the gap, the gap (d) values decrease, indirectly increasing the total pressure drop in the flow channel. If the force produced by MR damper has a correlation with the pressure drop that occurs between the before and after states of the MR valve, the increase of pressure drops means the increase of damping force. In addition, the difference in the peak force before and after the long-term operation in the off-state condition (refer to Figure 7) can be considerably higher than in the on-state condition (refer to Figure 5), and can be explained in the steady state model. The peak force in the off-state condition is mainly affected by $∆P_{viscous}$, where the channel gap, d, is raised to the power of three. 

### 3.3. Surface Roughness

**Figure 9:**
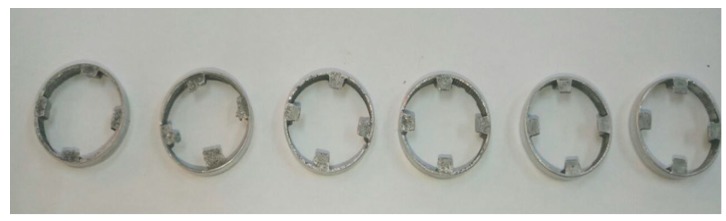
Spacer after long-term operation.

In this work, the wear effect of the device due to the impingement of iron particles is also investigated by visual observation on the surface of some MR valve components. Figure 9 shows the spacers after 170,000 cycles of operation. Some dented and uneven surfaces were observed in each spacer. The main reason for this problem is the clotting of the magnetic particles at the component surfaces, which are directly in contact with the fluid as a flow path. This eventually creates obstacles for the smooth flow motion of MR fluid. In this case, a higher force is required for the MR damper piston to move inward and outward [21]. 

### 3.4. Rheological Properties

**Figure 10:**
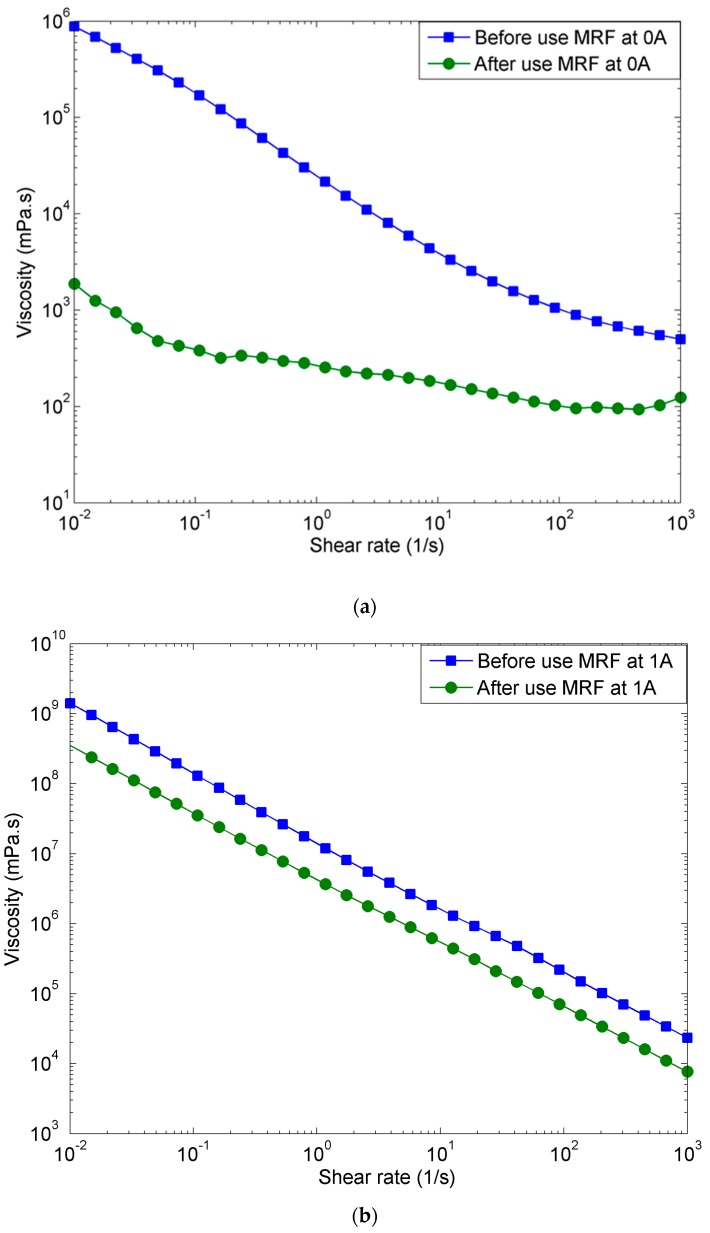

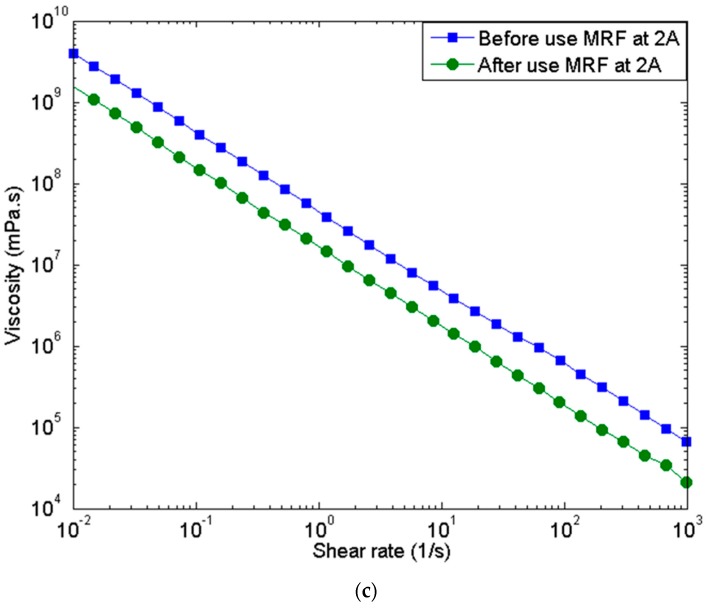
Shear rate-viscosity of MR fluid before and after the operation (**a**) without applied current, (**b**) at 1 A and (**c**) at 2 A of applied current.

In this work, the rheology tests are conducted using MCR 302, Anton Paar Companies, Graz, Austria, under two different conditions: on- and off-states. The results from the rheological tests for the off-state (without applied current) and for the on-state (at 1 A and 2 A of applied current) conditions are shown in Figure 10. Figure 10a shows the graph of viscosity versus shear rate for the off-state condition. The results show that the viscosity decreases with increasing shear rate before and after the 170,000 cycles, for both the off and on cases. In particular, for the off-state experiment, the initial viscosity for the fluid before use is 1 kPa s with 0.01 s^−1^ shear rate and, after 1000 s^−1^ shear rates, the viscosity decreased to 1 Pa s. For the same off-state experiment, the after-use MR fluid, that is, the MR fluid that has been subjected to 170,000 cycles, has an initial viscosity of 1 Pa s for 0.01 s^−1^ shear rate, and this value is further decreased to 0.1 Pa s for 1000 s^−1^ shear rates. There is a large difference in viscosity between new and used MR fluid, especially at the initial shear rates of 0.01 s^−1^. Indeed, after being subjected to the 170,000 cycles, the MR fluid exhibits an initial viscosity of 1 Pa s, which is 1000 times lower than what it exhibits before the cycles: 1 kPa s at a shear rate of 0.01 s^−1^. However, a smaller difference in the viscosity is observed for 1000 s^−1^ shear rates; the viscosity of the used MR fluid (0.1 Pa s), the fluid subjected to the 170,000 cycles, is 10 times lower than its viscosity before the long cyclic operation (1 Pa s).

Furthermore, Figure 10b,c show the viscosity of the MR fluid before and after the long operation in the on-state condition. A similar pattern to the results shown in Figure 10a is observed. From the results shown in Figure 10b, the initial viscosity of the MR fluid is identified as 1000 kPa s for 0.01 s^−1^ shear rate. It then decreases to 100 Pa s for 1000 s^−1^ shear rates. In this on-state condition of 1 A, the viscosity of the before-use MR fluid is about 10 times higher than the viscosity of the after-use MR fluid either for 0.01 or 1000 s^−1^ shear rates. A similar pattern is observed for 2 A magnetic field, as shown in Figure 10c, with higher values of viscosity compared to 1 A magnetic field. Figure 10c shows that the initial viscosity of the MR fluid is 10,000 kPa s, with 0.01 s^−1^ shear rate, and that it decreases to 100 Pa s for 1000 s^−1^ shear rates. In brief, the viscosity decreases along with the increase of shear rate for both cases, for before- and after-use MR fluid. However, the viscosity for the on-state conditions decrease in a manner that is inversely proportional to the shear rate, while this is not the case for the off-state condition. For the on-state condition, the viscosity of the MR fluid increases, with increasing applied currents. 

**Figure 11:**
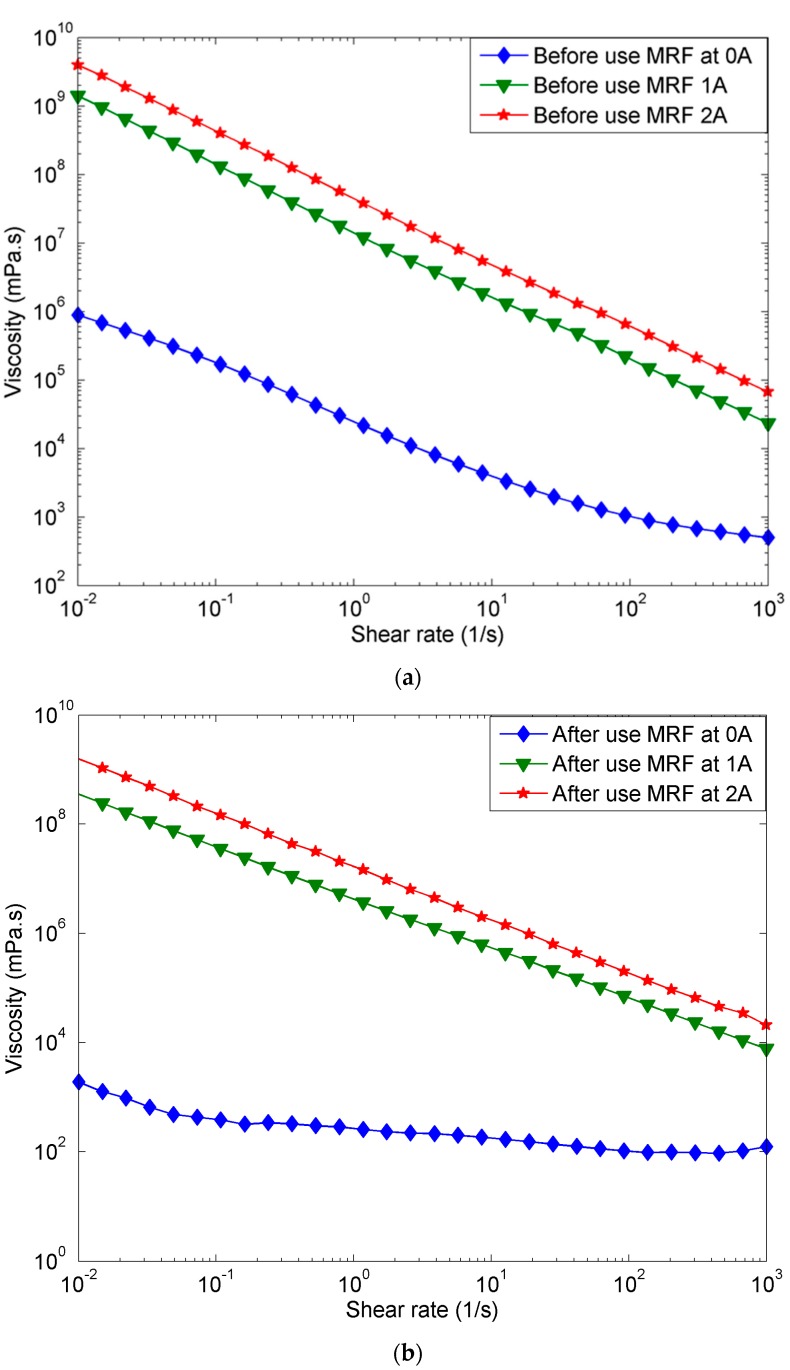
Shear rate-viscosity of (**a**) before-use MR fluid at 0 A, 1 A, and 2 A of applied currents, (**b**) after-use MR fluid at 0 A, 1 A, and 2 A of the applied currents.

Figure 11a compares the viscosity vs shear rate for MR fluid before the long-endurance test for off and on conditions, while Figure 11b compares these same parameters for MR fluid that has experience the long-endurance test. As a general trend, the viscosity of MR fluid decreases as the shear rate increases for both off- and on-state conditions. For instance, the relationship between the viscosity and shear rates in the presence of current is inversely linear, while the relationship under off-state condition (0 current) shows an inverse exponential pattern. Indeed, the difference in viscosity of MR fluid with 1 A is approximately 1000 times higher than the viscosity at 0 A. Meanwhile, the viscosity of the after-use MR fluid with 1 A is 1,000,000 times higher than the viscosity at 0 A. In brief, the results of viscosity of MR fluid after the long-term operation are considerably lower compared to the viscosity of the initial MR fluid for both on- and off-state conditions. The decrease in viscosity especially after long-endurance use of the MR fluid is due to the reduction in the number of magnetic particles in the MR fluid as time goes on. After the long-term operation with the continuous load of the magnetic field, the magnetic particles are deposited on the MR valve (refer to Figure 8), confirming that the MR fluid after the long operation has a lower number of magnetic particles, compared to the condition before the operation. This lower content of magnetic particles in the MR fluid results in a decrease in viscosity at both the off- and on-state conditions.

It is known that the rheological properties of MR fluid are affected by particle size and volume fraction [22], while the MR fluid viscosity is greatly affected by the magnetic field and magnetic particles [23]. In fact, the viscosity of the material, before and after the long operation, significantly changed. The magnetic particles are evenly mixed in the carrier fluid of the MR fluid inside the damper for the first few strokes, before sedimentation occurs inside the damper [15], affecting the content of MR fluid particles. With further operation of the system, sedimentation may lead to a smaller volume of MR particles, and a resulting lower viscosity of the MR fluid. There are several other possibilities for the lower viscosity. In this research, for example, the MR fluid was deposited at the valve when the fluid was forced by high pressure. The deposit of MR particles would agglomerate into a paste-like phase that remained in contact with the valve. Therefore, a smaller number of magnetic particles remained in suspension, contributing to the smaller composition ratio of particles to carrier fluid. In other words, the sample of MR fluid after the operation contained a smaller number of magnetic particles compared to the number of magnetic particles in the initial or before-use MR fluid. Consequently, the viscosity of MR fluid decreased at the end of the long-term operation. The study by Cvek et al. [24] revealed that the number of magnetic particles in MR fluid affects its viscosity. It showed that the greater concentration of magnetic particles cause higher shear stresses and, eventually, this higher shear stress causes higher viscosity [24]. However, this result is different with the previous studies conducted by Carlson et al. [11], who claimed that the viscosity of the MR fluid increased as the system’s operating time extended.

The meandering MR valve geometry, which is the combination of multiple annular and radial gaps, increases the path length of MR fluid flow [21]. This special geometry configuration consequently makes the magnetic particles and the carrier fluid easy to separate. Furthermore, the material of the valve itself, is magnetic, and is assumed to have remnants of a magnetic field, even though the operation has already finished. Another possibility is the applied mode operation. The principal operation of the damper commonly uses flow mode operation. Hence, in this particular MR valve, studies focused on flow mode operation. The magnetic particles produced an oxide layer at the outer surface of the particles as a result of the oxidation process between the iron particles and the surrounding environment. In addition, the magnetic particles of the MR fluid have a special characteristic, which is the capability to segregate among the particles themselves, and easily sediment in a certain area. There are multiple reasons behind the segregation of magnetic particles, such as shear-induced migration, gravity force, and centrifugal forces [25]. The segregation of the particles can noticeably change the volume fraction of the MR fluid from the initial condition.

### 3.5. Morphological Observation

**Figure 12:**
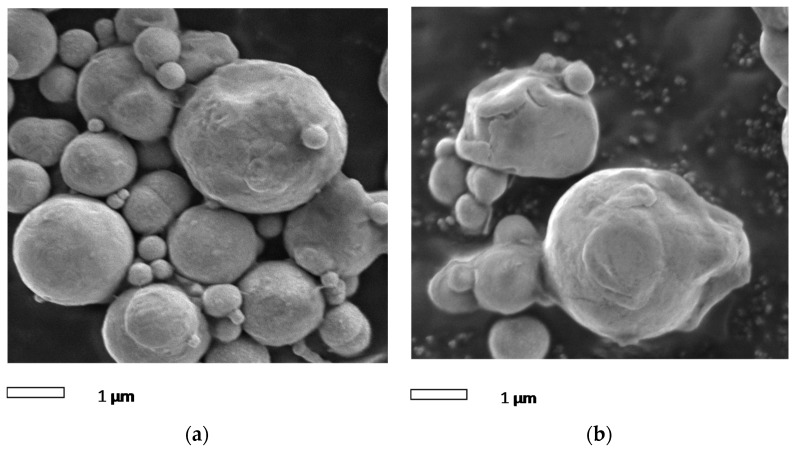
SEM images of magnetic particle of MR fluids (**a**) before and (**b**) after the long-term operation.

SEM micrographs of the magnetic particles extracted from MR fluid, before and after the long-term operation, are shown in Figure 12. The initial morphology of the magnetic particles before the operation is quite similar for all particles and present a spherical shape (Figure 12a), with the mean size of 1 to 5 μm. However, after the long-term operation, the magnetic particles deteriorated, both in shape and size, as presented in Figure 12b. The magnetic particles’ shape changed to irregular, and they also grew bigger in size. This might be due to the surface interaction between magnetic particles and the MR valve during the dynamic testing. In particular, during the dynamic testing, the MR fluid experienced an environment with high temperatures and elevated pressures. This high temperature leads to the welding of particles, increasing their size and changing their shapes. This similar condition has been reported by previous researchers [26]. Carlson et al. [11], stated that the oxidation process occurred during the operation, and resulted in the formation of an oxide layer surrounding the magnetic particles. This oxide layer may come from the composition of native oxides, carbides, and nitrides, that might result from the reaction of the material with the environment.

### 3.6. Oxidation

Oxidation of magnetic particles in the MR fluid becomes an obstacle in long-term operation of MR dampers, due to its impact on the behavior of MR fluid. Normally, the addition of additives to MR fluid should be considered to reduce the effect of oxidation [27,28,29,30]. The energy dispersive X-ray spectroscopy (EDX) measurement of the magnetic particles, before and after the operation, was investigated. It is observed that oxygen increased from 0.75% to 1.75%, indicating that the oxidation affected the MR fluid iron particles during the long operation. This finding confirms the study conducted by Desrosiers et al. [14], who worked on slippage clutch systems. Another study conducted by Plachy et al. [31] investigated the impact of thermal and chemical oxidative processes of carbonyl iron particles on MR performances, and found that the oxidized particles showed lower values of the yield stress that then affect the viscosity of MR fluid. Although the rheological and morphological properties of MR fluid have been shown to impact long-term operation, it is, however, still not clear which factor affects MR fluid performances, magnetic particles, or the carrier fluid more. Thus, further investigation in these areas are important, especially on the significant characteristics of carrier fluid and magnetic particles to the MR fluid performances in long-term operation. 

## 4. Conclusions

In this study, the rheological characteristics of the commercial MR fluid used in MR dampers has been investigated after long-term operation. The performance of MR fluid was confirmed to change after long cyclic loading applied by a fatigue dynamic machine. Firstly, it was observed that the damping force increases after long-term operation under both on- and off-states. It was observed that the size and shapes of magnetic particles in the MR fluid had changed after long-term operation. The damping force of the MR damper increased by about 44%, compared to the initial force for the on-state condition and 90% for the off-state condition after 170,000 operating cycles. Besides, the viscosity of the MR fluid was reduced for both on- and off-states, because of IUT and oxidation phenomenon in MR fluid during the long operation. The magnetic particles of the MR fluid also changed considerably in both shape and size after the operation. Regarding the rheology of the MR fluid, before it is subjected to long-term operation, it has a viscosity of 1 kPa s with 0.01 s^−1^, while after experimental work, the MR fluid’s viscosity was 1 Pa s with 0.01 s^−1^ shear rate. The used MR fluid had 1 Pa s viscosity, which is 1000 times lower than the respective initial viscosity of non-used MR fluid, which is approximately 1 kPa s. This is because a smaller number of magnetic particles are retained after the long-term operation, compared to the amount of the magnetic particles present on the initial MR fluid. The authors believe that these preliminary results would be very useful for further consideration in MR-based devices’ design. This is because almost all operating conditions of the devices will be cyclic loading. Therefore, to achieve better reliability for MR-based devices, the prediction of MR fluids’ degradation must be investigated.

## Figures and Tables

**Table 1:** Principal properties of MRC-C1L.

Appearance	Dark Grey Liquid
Solid Weight Percentage (wt %)	77–80
Density (g/m^3^)	2.75–2.95
Shear Stress (kPa @ 1500/s 570 mT)	68.0 ± 7
Viscosity (Pa s)	0.106 + 0.020
Flash Point (°C)	>140
Operating Temperature (°C)	−40 to +140
Sedimentation Stability (vol %/30 days)	4.00

**Table 2:** Magnetic fields in magnetorheological (MR) valve.

Current (A)	Flux Density in MR Valve Areas (T)					
	Annular 1	Radial 1	Radial 2	Radial 3	Radial 4	Annular 2
1	0.63	0.84	0.84	0.84	0.84	0.63
0.8	0.52	0.72	0.72	0.72	0.72	0.52
0.6	0.38	0.58	0.58	0.58	0.58	0.38
0.5	0.31	0.50	0.50	0.50	0.50	0.31
0.4	0.24	0.43	0.43	0.43	0.43	0.24
0.2	0.13	0.24	0.24	0.24	0.24	0.14
